# Power spectral aspects of the default mode network in schizophrenia: an MEG study

**DOI:** 10.1186/1471-2202-15-104

**Published:** 2014-09-05

**Authors:** June Sic Kim, Kyung Soon Shin, Wi Hoon Jung, Sung Nyun Kim, Jun Soo Kwon, Chun Kee Chung

**Affiliations:** MEG Center, Department of Neurosurgery, Seoul National University Hospital, Seoul, South Korea; Sensory Organ Research Institute, Seoul National University, Seoul, South Korea; Department of Psychiatry, Seoul National University College of Medicine, Seoul, South Korea; Department of Neurosurgery, Seoul National University College of Medicine, 28 Yeongun-dong Jongro-gu, Seoul, 110-744 South Korea; Department of Brain and Cognitive Sciences, Seoul National University College of Natural Sciences, Seoul, South Korea

**Keywords:** Default mode network, Magnetoencephalography, Power spectral density, Alpha, Schizophrenia

## Abstract

**Background:**

Symptoms of schizophrenia are related to deficits in self-monitoring function, which may be a consequence of irregularity in aspects of the default mode network (DMN). Schizophrenia can also be characterized by a functional abnormality of the brain activity that is reflected in the resting state. Oscillatory analysis provides an important understanding of resting brain activity. However, conventional methods using electroencephalography are restricted because of low spatial resolution, despite their excellent temporal resolution.

The aim of this study was to investigate resting brain oscillation and the default mode network based on a source space in various frequency bands such as theta, alpha, beta, and gamma using magnetoencephalography. In addition, we investigated whether these resting and DMN activities could distinguish schizophrenia patients from normal controls. To do this, the power spectral density of each frequency band at rest was imaged and compared on a spatially normalized brain template in 20 patients and 20 controls.

**Results:**

The spatial distribution of DMN activity in the alpha band was similar to that found in previous fMRI studies. The posterior cingulate cortex (PCC) and lateral inferior parietal cortex were activated at rest, while the medial prefrontal cortex (MPFC) was deactivated at rest rather than during the task. Although the MPFC and PCC regions exhibited contrasting activation patterns, these two regions were significantly coherent at rest. The DMN and resting activities of the PCC were increased in schizophrenia patients, predominantly in the theta and alpha bands.

**Conclusions:**

By using MEG to identify the DMN regions, predominantly in the alpha band, we found that both resting and DMN activities were augmented in the posterior cingulate in schizophrenia patients. Furthermore, schizophrenia patients exhibited decreased coherence between the PCC and MPFC in the gamma band at rest.

## Background

Schizophrenia is a psychotic disorder that alters patients’ perception, thought processes, and behavior expressed by hallucinations, delusions, disorganized speech, social withdrawal, and various cognitive deficits [1]. A deficit in self-monitoring that leads to the experience of endogenous thought or inner speech as alien is the core of the disorder [2]. The default mode network (DMN) is regarded as a network reflecting self-monitoring and stimulus-independent thought, and is thus of particular interest in schizophrenia research [3]. In addition, structural [4] and functional abnormalities [5, 6] in schizophrenia patients have been reported in various neuroimaging studies. For example, schizophrenia patients were recently reported to exhibit significant cortical thinning in the prefrontal cortex, the anterior and posterior cingulate cortex (PCC), and the superior temporal and parietal regions [4]. In functional studies, dysfunctional connectivity between the frontotemporal brain regions was suggested to be a central feature of schizophrenia [5, 6]. Parts of these regions are also important for the resting state network. Therefore, aberrant DMN activity may be observed in schizophrenia patients.

Resting-state brain activity in schizophrenia patients has been previously investigated. Schizophrenic patients show augmented low-frequency power and diminished alpha-band power according to the degree of negative symptoms in the resting state [7]. In an fMRI study, the spatial location and temporal frequency of the DMN were shown to be abnormally altered in schizophrenic patients [8]. Nevertheless, the relationship between schizophrenia and abnormal electrophysiological activity at resting state remains unclear.

The DMN is known to be active during the resting state of brain function [9, 10]. This network, including the precuneus, PCC, medial prefrontal cortex (MPFC), and medial/lateral/inferior parietal cortex, is task-nonspecifically deactivated during goal-directed activity. The network also exhibits significant functional connectivity characterized by very low frequency temporal synchrony in the resting state [11–13]. The DMN has been described as a task-negative network, indicating that there is an apparent antagonism between its activation and task performance. Additionally, the DMN is temporally anticorrelated with the task-positive network [14].

The properties of this network are typically reported using conventional neuroimaging modalities such as functional magnetic resonance imaging (fMRI) [12] and positron emission tomography (PET) [10]. However, because the sampling rate of fMRI and PET is generally more than a second, it is difficult to use these modalities to interpret rapidly changing brain activity.

There is some evidence of a correlation between the slowly changing blood oxygen level dependent (BOLD) activity and fast electrical neuronal activity. Furthermore, using simultaneous fMRI and electroencephalography (EEG) recordings, increasing reports of correlations between DMN and theta [15], alpha [16], beta [17], and gamma [16] activities have been made. In addition, other invasive studies using local field potential have reported that electrical activity in the gamma band is reduced in the DMN nodes on the initiation of an attention-demanding task [18, 19]. A recent review study showed evidence for a general trend towards a negative correlation between BOLD and low frequency MEG oscillations, and a concomitant positive correlation between BOLD and high frequency MEG oscillations [20]. Another study, using simultaneous fMRI-EEG recording, found a strong correlation between spontaneous BOLD response in the left parahippocampal gyrus and delta power from the anterior cingulate cortex [21]. Furthermore, the authors reported that BOLD responses in the supplementary motor cortex and precuneus were respectively correlated with low and high beta activity in the PCC, suggesting that the separate frequency domains may originate in subsets of the DMN.

In a recent magnetoencephalography (MEG) study, beta and gamma band activation in the resting state were observed during working memory tasks [22]. Another MEG study using a seed-based correlation analysis showed that neural oscillations mediate functional connectivity between the resting state networks reported in fMRI studies [23]. Maldjian et al. also reported that hubs of the MEG networks were predominantly symmetric and centered around the midline, with some resemblance to fMRI networks in an MEG study using graph theoretical analysis [24]. Furthermore, an EEG study using independent component analysis (ICA) revealed functional connectivity within the resting state network [25], while some task-positive studies using ICA also reported spatial correlation with BOLD networks [26, 27]. However, EEG frequency bands and many of the EEG/MEG resting studies are focused on correlations with BOLD signals or connectivity approaches. Thus, it is necessary to identify DMN activation with respect to the electrophysiological activity itself. This would allow the use of neurophysiological DMN activation for the diagnosis of various neurological and psychiatric diseases (e.g., schizophrenia in the present study). For example, there have been several trials that have interpreted DMN activation for neurological and psychiatric disorders such as epilepsy [28] and attention-deficit/hyperactivity disorder [29].

The aim of the present study was to identify brain regions representing the “default mode” using MEG, and to investigate the functional abnormalities in these regions in patients with schizophrenia. We hypothesized that components of MEG activity represent conventional DMN, and that the DMN activity of schizophrenia patients would differ from controls. To examine the hypothesis, we evaluated the difference between the power spectral densities during the resting state and performing a task in the cortical surface model, and also compared between healthy participants and schizophrenic patients.

## Methods

### Ethics statement

The Institutional Review Board (IRB) of the Seoul National University Hospital approved the study. Written informed consent was obtained from all participants or legally authorized representatives prior to experiments, according to the local IRB rules and local laws. We assessed the capacity of patients to provide consent for participation in this study on a case-by-case basis. Patients without the capacity to provide informed consent were not included in the study. For any patient who did not have legal status to provide informed consent, their guardian authorized the research by signing the consent form.

### Participants

Twenty schizophrenia patients (mean age 22.8 years, standard deviation 3.9 years, range 17–33 years; 4 female) and 20 healthy controls (mean age 22.1 years, standard deviation 2.0 years, range 18–25 years; 6 female) volunteered to participate in this study and provided written consent. We used most of the participants from a previous study that investigated pre-attentive auditory deficits in schizophrenia and its ultra-high-risk group [30]. In the previous study, 18 and 15 participants were analyzed as the control and schizophrenia groups, respectively. In the present study, we added two and five more participants to the control and schizophrenia groups, respectively, to the participant pool of our previous study. Table 1 provides the demographic and clinical data for each group.

**Table 1:** **Demographics of participants**

	Normal control		Schizophrenia		Analysis		
	(N = 20)		(N = 20)				
	Mean	SD	Mean	SD	F or ***χ*** 2	df	P
Male/Female	14/6		16/4		0.90	2	0.637
Age (years)	22.06	2.04	22.80	3.91	0.80	2	0.459
Education (years)	14.06	1.16	12.30	1.83	3.90	2	0.028
Handedness*	11.39	1.65	10.40	3.10	1.40	2	0.266
Parental SES	2.83	0.98	2.40	0.70	1.42	2	0.253
IQ	107.50	17.13	99.20	6.91	2.91	2	0.066
GAF	90.89	2.99	64.30	11.67	113.42	2	0.000
PANSS			54.20	12.89			
Duration of illness (years)			5.83	3.03			
Schizophrenia: Paranoid subtype			10				

*Scores of Annett Hand Preference Questionnaire (AHPQ) consisting of 12 questionnaires.

Schizophrenia patients were diagnosed using the Structured Clinical Interview for Diagnostic and Statistical Manual of Mental Disorders, Fourth Edition Axis I Disorders (SCID-IV) and were assessed using the positive and negative syndrome scale (PANSS) and global assessment of function (GAF) on admission into the study. All patients were receiving pharmacotherapy with atypical antipsychotics and had a stable clinical status over the previous year. 20 controls were recruited so that their age, sex, and Intelligence Quotient (IQ) were similar to those of the schizophrenia patients. Control participants were screened using the SCID-IV-Nonpatient Edition (SCID-NP). Exclusion criteria included past or current Axis I diagnoses or any first- to third-degree biological relatives with a psychiatric disorder. Exclusion criteria for all participants included a lifetime diagnosis of substance abuse or dependence, neurological disease or head injury, evidence of medical illness with documented cognitive sequelae, sensory impairments, intellectual disability (IQ < 70), or musical training within the previous 5 years.

### MRI acquisition

All participants were scanned in the axial plane using a 1.5 T MR unit (Siemens Avanto, Germany) and T1-weighted 3-D magnetization-prepared rapid-acquisition gradient-echo sequence for head images. The acquisition parameters were as follows: TE/TR = 4.76/1160 ms, flip angle = 15°, FOV (field of view) = 230 mm, voxel size = 0.45 × 0.45 × 0.9 mm. From a visual inspection, all scans were judged to be excellent without obvious motion artifacts, signal loss, or gross pathology.

### MEG acquisition

All participants underwent MEG examination during the resting state and while performing a task maintaining alertness. In both states, the participants were instructed to stay still in the whole-head MEG system (VectorView™, Elekta Neuromag Oy, Helsinki, Finland) in the MEG Center at Seoul National University Hospital, which consists of 306 channels arranged in triplets of two planar gradiometers and one magnetometer. The sampling frequency was 1001.6 Hz, and the signal was filtered by an analog filter in the range of 0.1–200 Hz. A bipolar electrooculogram (EOG) and an electrocardiogram were simultaneously recorded during every measurement to monitor eye blinking, eye movement, and cardiac artifacts. The location of the participant’s head with respect to the sensors was determined by measuring four head position indicator coils that were sparsely attached to the scalp and produced a magnetic field. Before each recording session, the positions of the head coils were digitized in relation to three anatomical landmarks (i.e., nasion and left and right preauricular points). To align the MEG and MRI coordinate systems, three-dimensional digitization was performed (FASTRAK, Polhemus, Colchester, VT, USA).

Every participant received the same recording protocols for MEG. A participant performed one task session (6 min) after recording one resting session (2.5 min). In the resting session, participants were seated on a measurement chair for MEG, and were instructed to keep their posture still and their eyes open and fixed on a small cross-hair in the middle of the screen during an approximately 150 s continuous recording session.

In the task session, participants were instructed to seek Wally in a figure projected to a screen in front of the subject. A figure was randomly selected from a picture book called “Where’s Wally?” The next figure was presented whenever the participant successfully found Wally and pressed the response button. During the task session, auditory stimuli consisting of 1000 Hz pure tones (80 dB, 10 ms for rise and fall) with different durations of 50 and 100 ms were simultaneously provided. A tone with a 50 ms duration was used as a standard stimulus, and a tone with a 100 ms duration was used as a deviant stimulus. Two tones with different durations were pseudo-randomly presented to the participants with a 300 ms stimulus onset asynchrony at a rate of 81.8% and 18.2%, respectively. The stimuli were presented using tubular insert earphones with the STIM2 system (Neuroscan, El Paso, TX, USA).

### Preprocessing

To construct the source image of the gray matter, a cortical surface model was made for every participant using the CLASP algorithm, which reconstructed the inner and outer interfaces of the gray matter with 40,962 vertices for each hemisphere [31]. A mid surface between the inner and outer interfaces was generated by averaging the corresponding points of the two interfaces. Additionally, the vertices consisting of the mid surface were down-sampled to maintain more than 10 mm intervals between points. The number of source points after the down-sampling was 4150 in the whole-brain model. The source images were reconstructed on the down-sampled points. After the source imaging, a linear interpolation method was used to display the image results on the surface.

After the MEG recording, a Maxwell filter, which separated brain-related and external interference signals, was first applied to reduce environmental and biological noise [32, 33]. In addition, the filtered signals were visually inspected to reject time intervals with excessive noise, muscle artifacts, eye blinks and eye movements.

### Source imaging

A lead field, which describes the sensitivity pattern of each MEG sensor [34], for 306 gradiometers and magnetometers, was computed on the points with a spherical head model. The center of the sphere was fit from manually selected inner skull points. If *B* is the vector of simultaneous MEG signals (signal vector), the forward solution has a simple linear form, given by


where *S* indicates a vector of dipole component strengths which includes brain activity, *n* is the measurement noise, and *A* denotes a lead field generated from the points representing the gray matter. A spherical head model was used for volume conduction.

The signal at each polygonal point consisted of two orthogonal dipole components tangential to the surface of a spherical head model. Therefore, the source model consisted of two source vectors for each point. In this study, we used a standardized low resolution brain electromagnetic tomography (sLORETA) algorithm for source modeling [35]. The modified pseudo-statistics of sLORETA were used to analyze the absolute activation at each point. The weight matrix at the *j*-th point, *W*_*j*_, is given by


where *I* is the identity matrix, *W*_*j*_ is a row vector, and *α* is a regularization parameter. For any matrix *M*, *M*^*+*^ denotes its Moore-Penrose pseudo-inverse [36]. Note that the denominator indicates the standard deviation of the estimated current density.

The current density at the *j*-th point, *S*_*j*_, is given by


### MEG analysis

Spectral analysis was performed on the fast Fourier transform (FFT). FFT was applied to the raw MEG signals, and then the FFT data were localized on the source space. Theta, alpha, beta, and gamma band activities were separated in the source space. To do this, the spontaneous MEG data and the task data were evenly divided every 1 s without an overlap. Among divided segments, clean segments were selected without eye-blinking, eye-movement, and other excessive noises by manual inspection of the MEG and EOG signals. The number of selected segments for the resting data ranged from 110 to 135 in our participants while the number of selected segments for the task data ranged from 140 to 180. Note that there were more rejected segments in the task session than in the resting session due to a visual search paradigm. A Hanning window was applied to the segments, and the FFT was applied to each segment. The real and imaginary parts of FFT at each source point were separately estimated using the spatial filter matrix described in section Source imaging. The details of the method follow those described by Jensen and Vanni [37] and our previous study [38]. Compared with previous methods, we used the Fourier transform and sLORETA for source reconstruction instead of the wavelet transform [38] and minimum-current estimation [37], respectively. The Fourier transformed signals *S*^*k*^(*f*) were computed for each segment, *k*, with respect to the frequency of *f*. The real *S*^*k*^(*f*)_Re_ and the imaginary part *S*^*k*^(*f*)_Im_ of the Fourier transformed signal were then applied to the sLORETA filter resulting in the current distributions, *Q*^*k*^(*f*)_Re_ and *Q*^*k*^(*f*)_Im_, respectively. The absolute current estimates at the *j*-th source point for all the segments were average in the source space, as follows.


Finally, we divided the frequency spectrum into several frequency bands: theta (4–7 Hz), alpha (8–12 Hz), beta (13–30 Hz), and gamma (30–50 Hz).

There are two power spectral densities at each source location. Since two source activities are orthogonal, the power spectrum at each location can be combined using vector addition. The combined power at the *j*-th source location and a given frequency *f*, *P*_*j*_(*f*), is given by


where P_j_^(1)^ and P_j_^(2)^ indicate the power spectra of two dipole sources.

### Surface registration

Individual surface models were nonlinearly transformed into the template for the group analysis using a surface registration algorithm described in detail elsewhere [39, 40]. Briefly, the method performs a hierarchical deformable registration of the surfaces from a coarse to a fine scale to find the optimal vertex correspondence based on the feature field matching, combined with a regularization step that preserves the local surface topology. The mapping values on the individual cortical surfaces were then spatially normalized into the template. Statistical analysis was then performed with the spatially normalized sLORETA images.

### Statistical analysis

**Figure 1:**
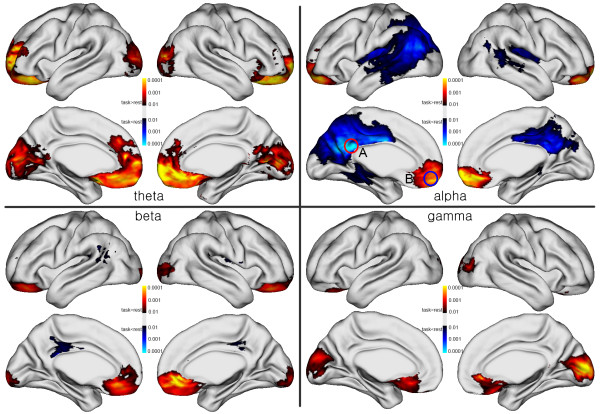
**The power difference maps in the control group between resting and at-task states.** Theta (left top), alpha (right top), beta (left bottom) and gamma (right bottom).

Global and regional differences in the power spectral density between the resting and task conditions were computed using an independent *t*-test after linear regression with covariates for age and gender. We selected age and gender as covariates because previous structural and functional studies reported age and gender differences. However, in the results, age and gender did not predict variance in the measures of interest. Significant effects were reported when they passed a whole-brain false-discovery rate (FDR) correction for multiple comparisons at P < 0.01. The FDR refers to the expected proportion of falsely declared associated points among all points that are declared associated [41]. The results were mapped onto the surface template model where statistically significant results were expressed in color (Figure 1).

**Figure 2:**
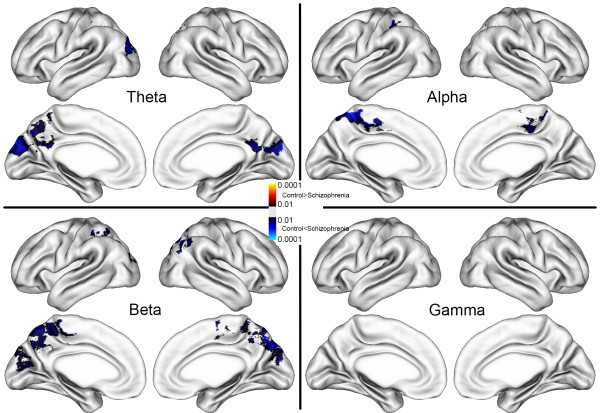
**Regional difference in DMN activation between the control and schizophrenia groups in the delta (top left), theta (top right), alpha (bottom left), and beta (bottom right).** With a permutation *t*-test, only significant vertices (p < 0.01) were colored.

**Figure 3:**
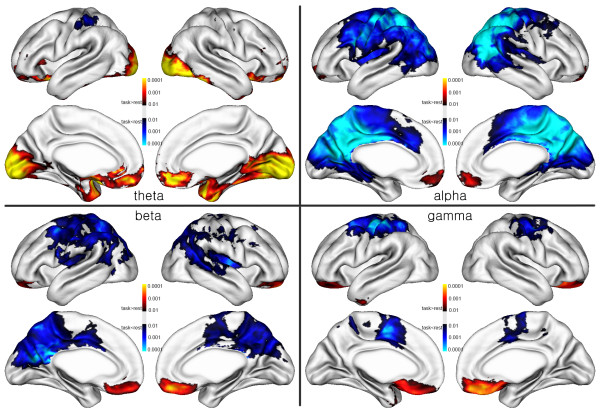
**The power difference maps in the schizophrenia group between resting and at-task states.** Theta (left top), alpha (right top), beta (left bottom) and gamma (right bottom).

Global and regional differences between schizophrenia and control groups in the DMN activity (i.e., the difference in the power spectral density between the resting and task conditions) were also analyzed (Figure 2). We used permutation tests to confirm that the significant changes were not purely by chance for multiple comparisons, as follows [42]. To accomplish this, participants were randomly assigned to groups across 10,000 new randomized analyses at each vertex and the number of significant results (i.e., the power spectral density at any vertex that significantly differed between groups at a threshold of p = 0.01) that occurred in the real test for group differences was compared with the null distribution of significant results that occurred by chance. The permutation analysis was estimated on single vertices. In the resulting images, only significant vertices (p < 0.01) are shown in Figures 1, 2, and 3.

**Figure 4:**
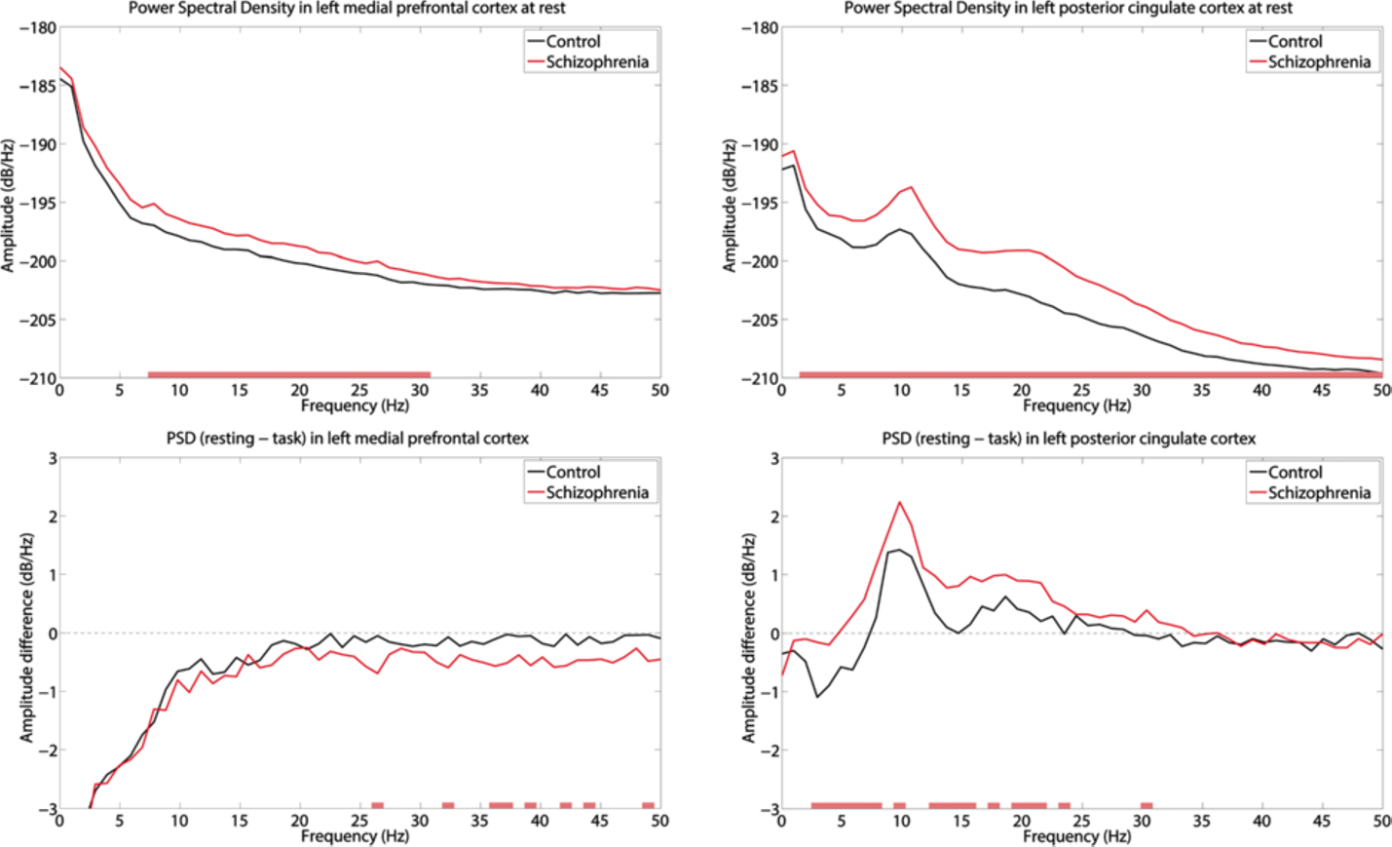
**Power spectral density of the resting state at the left MPFC (top left) and left PCC (top right), and the difference power between the resting and at-task states at the left MPFC (bottom left) and left PCC (bottom right).** Significantly different frequencies (p < 0.01) between the control and schizophrenia groups are indicated as small red bars at the bottom of each graph.

**Figure 5:**
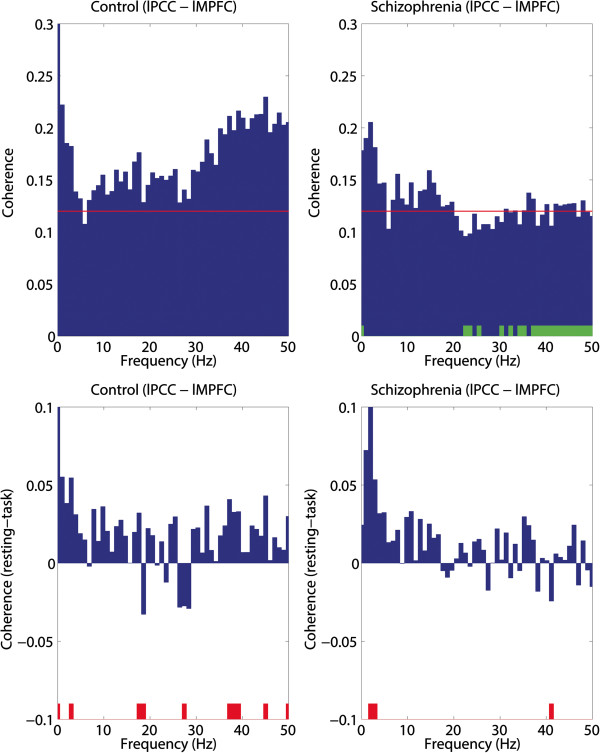
**Coherence spectra between the medial prefrontal cortex and posterior cingulate cortex.** Coherence at rest (upper row) and coherence difference between the resting and at-task (lower row) states for the control (left) and schizophrenia (right) groups. In the coherence spectra (upper row), the confidence level (p = 0.01) is drawn as a red line. Significant differences between the control and schizophrenia groups are marked with small green bars in the schizophrenia graph (upper right). In the coherence difference spectra (lower row), significant differences between the resting and at-task conditions are marked with small red bars.

The regional power spectra were extracted from the locations showing the maximal DMN activity in the PCC and MPFC. The power spectrum and DMN activity were then compared with an independent *t*-test (Figure 4). We also analyzed the coherence between two selected locations and the coherence difference between conditions (Figure 5). Coherence, *Coh*_*XY*_(*f*), between source activities on X and Y was calculated according to the following equation:


where *F*_*XY*_(*f*) is the cross-spectrum for the source activities of *X* and *Y* at a given frequency *f. F*_*XX*_(*f*) and *F*_*YY*_(*f*) are the respective power-spectra of the source activities of *X* and *Y* at the same frequency. Coherence is bounded between 0 and 1, where 0 indicates the complete absence of a linear relationship and 1 represents a perfect linear relationship between *X* and *Y* at frequency *f*.

We selected a voxel showing the strongest activity at each ROI as a representative location. At the selected voxel, the time series of spontaneous MEG data were distributed in the two-dipole space. We estimated the principal vector using singular value decomposition. Two dipole activities were projected onto the principal vector, and the coherence analysis between two ROIs was estimated using the projected signals.

## Results

### Default mode network in MEG

Figure 1 shows the difference maps according to the frequency bands between the resting state and task periods in the control participants. After a *t*-test using permutation, p-values were corrected by FDR. The alpha band frequencies increased during the resting state, while theta and gamma band frequencies increased during the task period. The mapping pattern of beta band activity was similar to that of the alpha activity, but with a smaller spatial extent than that of the alpha. Most of the frequency bands showed similar spatial patterns. The PCC extending dorsally into the precuneus, dorsomedial prefrontal cortex, and lateral cortex were involved in the difference maps. Previous PET and fMRI studies have established that these regions form part of the default mode network [10]. Interestingly, the spatial pattern of the alpha difference map was the most similar to the resting-state network of the fMRI and PET studies. The default mode activity in the alpha band was apparent in the PCC, precuneus, and lateral cortex. However, the activities were reversed in the ACC and MPFC, while we could not find any frequency band showing positive DMN activity in the MPFC.

In the schizophrenia group, the spatial map was similar to that of the control group. However, the overall DMN activity was stronger than that of the control group (Figure 3).

### Regional difference in DMN activity between groups

The regional differences in the DMN activity between the control and schizophrenia groups, depending on the frequency band, are shown in Figure 2. Schizophrenia patients generally showed increased DMN activities in the theta, alpha, and beta bands. The main locations were similar to each other even though the extent of activity was different. These hyperactivities were found mainly in the posterior region which showed DMN activity in the alpha and beta bands, while there was no significant difference in the gamma band.

### Power spectra at the cingulate and medial prefrontal cortices

In the alpha band difference shown in Figure 1, the posterior and anterior medial cortices show reciprocal DMN activities. The posterior part increased while the anterior part decreased during the rest period compared with the task period. We examined the regional power spectral density in these two regions, which were identified from the difference map of the controls (Figure 1). Figure 4 shows the power spectral density at the PCC and MPFC. To determine this, we selected two grid points showing the strongest alpha difference in both regions. The selected points are also shown in Figure 1 as circles. The “A” circle represents the PCC, while the “B” circle indicates the MPFC region. As shown in the upper panels of Figure 4, the schizophrenia group had greater power in both regions than that in the control group at rest. The DMN activity can be defined as activity that is more decreased during a goal-oriented task than at rest. We refer to the difference in activity between rest and task as the DMN activity. DMN activity was strong, indicated by the greater activity at rest rather than at task. Additionally, the DMN activity in the PCC was significantly enhanced in schizophrenia patients in the alpha, beta, and theta bands. The group difference of the power spectra in the MPFC was partly significant in the gamma band, while the other bands were not significantly different. This resulted in the lack of a significant group difference in the DMN activity in the anterior region of the brain (Figure 2).

Next, we performed correlation analysis between PANSS and power spectra of the two regions. There was a significant correlation (p < 0.05) between the positive syndrome scale and the DMN activity of gamma power in the MPFC. Note that there were also significant group differences in gamma (left lower panel in Figure 4). However, we could not find a significant correlation in the PCC, and there was no significant correlation between clinical measures and power in the resting state.

### Connectivity analysis

In this study, the PCC and MPFC had opposite DMN activities although many previous studies using fMRI have reported a strong relationship between the two regions. To investigate the connectivity between the PCC and MPFC, we performed coherence analysis. Although the anterior and posterior parts showed contrasting DMN activity in the alpha band, the coherence analysis showed significant connectivity between the two regions during the resting state (upper row, Figure 5). A significant coherence level was represented by values above the 99% confidence level [43]. The coherence difference between the controls and schizophrenia patients at rest was calculated after applying Fisher’s z-transform to the square root of the coherence. Significant differences (p < 0.01) are marked with green bars on the graphs. The schizophrenia group showed a significantly decreased coherence in the gamma band. The coherence difference between the rest and task conditions (lower row, Figure 5) was slightly increased in most of the frequency bands during rest for each group.

## Discussion

### Default mode network in magnetoencephalography

The DMN is associated with intrinsic, ongoing brain activity. We revealed that the default mode network is also involved in various MEG frequency bands. In particular, the spatial distribution of the DMN brain activity in the alpha band was similar to the well-known DMN activation in fMRI studies [44]. Another recent EEG study using an ICA technique also reported spatial patterns of brain activity in the alpha band, which showed considerable overlap with the DMN [45].

Alpha oscillation is considered to be a stable idling rhythm in the mammalian brain. This rhythm appears during a wakeful and alert state with the eyes closed [46], and may act as a baseline for specific brain structures associated with the attentional system [17]. Alpha oscillation originates from the oscillatory post-synaptic potentials within the thalamocortical and corticocortical regions that persist in an attentive state [47].

In the PCC, the majority of frequency bands showed increased activity in schizophrenia patients. Many previous studies have reported a narrow band difference [7, 48]. While a channel-level analysis of EEG and MEG represents the activation of the whole brain, it may suffer from inaccurate discrimination of regional activation. Thus, it is possible that the power spectral analysis at a source location in our study might produce slightly different results from studies that use EEG electrodes. Owing to volume conduction, the brain signal on an electrode may represent the summed activity of brain sources. On the other hand, the normalization of power spectra between groups may be necessary to determine the differences in a specific frequency band. Our study focused on the absolute difference of power spectra between schizophrenia patients and controls, and normalization was performed for the DMN activity.

We found a significant correlation between positive syndrome scale and gamma power in the MPFC. This suggests that the MPFC is a key region for reflecting the clinical symptoms of schizophrenia, despite the lack of a group difference in the MPFC (Figure 2). However, the abnormal broad-band activity was not correlated with PANSS in either the PCC or the MPFC. Although the abnormal resting activity in schizophrenia was not correlated to the clinical measures, it may be activity that is specific to the illness.

In our previous study investigating event-related desynchronization (ERD) using MEG, the alpha ERD was diminished in schizophrenia patients [49]. The decreased alpha ERD has also been observed in many other studies using auditory tasks [50–54]. These studies have reported less post-stimulus alpha band activity in schizophrenia patients than in controls. However, the alpha ERD of those studies was computed using the brain response normalized by brain signals immediately prior to stimulation onset. Thus, the baseline of the study might not strictly be at resting state owing to the frequent stimulation sequence (1150 ms inter-stimulus interval). Therefore, these data suggest that there is a smaller gap between before and after the stimulus onset during the task session in schizophrenia patients compared with healthy controls. On the other hand, the results of the present study suggest that schizophrenia patients are highly activated even at rest, and that the power difference between resting and task is reduced. This abnormal activity may be a reflection of spontaneous symptoms of schizophrenia such as hallucinations and delusions.

In schizophrenia, we found that brain activity in the alpha and beta bands increased at rest in the MPFC, while brain activity at most frequencies was increased in the PCC. There have been inconsistent findings regarding changes in alpha and beta band activities in schizophrenia patients, with no significant difference [48], an increase [55–57], or even a decrease [7]. The majority of resting-state EEG and MEG studies in schizophrenia patients have used the eyes-closed task, in contrast to the eyes-open task used in the present study. Alpha activity can depend on whether the eyes are open or closed. Thus, the eyes-open condition may affect the DMN activity of the alpha band and the difference between the schizophrenia and control groups in our study. However, it is the most similar comparison to the resting-state fMRI and PET studies as these typically use an eyes-open task. While eyes-closed studies find decreased alpha activity [7], the present eyes-open findings indicate abnormally increased alpha and beta. This result implies that schizophrenia patients do not properly modulate alpha rhythms. Future studies examining eyes-closed and eyes-open alpha patterns in schizophrenia patients are of interest.

### Anti-DMN-activity of alpha between the PCC and MPFC

The brain activity in the alpha band may represent DMN activity in the PCC, precuneus, and parietal cortex but not in the MPFC. Although the entire contrast map of alpha oscillation between resting and task is similar to previous fMRI and PET studies [10, 12], the anterior and posterior parts of the brain show reciprocal activation. The posterior activation supports fMRI and PET studies that show increased brain activity at rest. Interestingly, in our study, the MPFC and anterior cingulate showed negative DMN activity irrespective of frequency band. This result is contrary to the conventional DMN activation in fMRI and PET studies, which is greater at rest than at task. This result might be due to the limited frequency band of 4–50 Hz used for the present analysis. Considering the differences in neuroelectricity versus hemodynamic responses and of relatively high versus low frequency bands, it is difficult to find an exact match of characteristics between EEG/MEG and fMRI/PET. In addition, DMN activity is known to be task-nonspecifically deactivated during a goal-oriented task in fMRI and PET. However, there are few studies that show this deactivation during a task in EEG and MEG. Further studies are required to identify the alternative activity of the anterior brain regions.

### Connectivity between the posterior cingulate and medial prefrontal cortex

Despite opposite activities between the anterior and posterior parts of the brain, we found significant coherence between the two regions at rest. These data support the findings that DMN regions are correlated with each other at rest [12, 13]. This connectivity suggests that the brain oscillation within DMN regions is correlated, from the slow fluctuation in fMRI to the fast oscillation in MEG. As this resting-state coherence slightly decreases during the task with a significant change at some frequencies, connectivity characteristics of fMRI and PET would be maintained in fast MEG activity. Nevertheless, this connectivity property does not indicate a correlated oscillatory activity in most of the frequency bands.

When we compared the resting coherence between participants, resting connectivity was significantly decreased in the gamma band in patients with schizophrenia. While there are a few reports of hyper-connectivity in schizophrenia [58, 59], there is also evidence of reduced connectivity [60, 61]. As the majority of previous connectivity studies used the slow fluctuation of fMRI, the results of the present study suggest aberrant high frequency connectivity at rest in schizophrenia. A recent study using MEG also found reduced gamma connectivity across the posterior medial parietal cortex in schizophrenia patients [62]. Another study also reported decreased imaginary coherence in schizophrenia patients [63]. Thus, this decreased gamma band connectivity might be a biomarker for schizophrenia.

We also found a significant slow wave (theta–lower beta) connectivity in both schizophrenia patients and control participants. Thus, long-range functional connectivity via coupling between the alpha and lower frequencies can remain intact in schizophrenia. However, in coherence comparisons, higher beta and gamma coherence over 20 Hz decreased in schizophrenia patients compared with controls. Some ECoG [64] and MEG [65] studies have reported that gamma activity is modulated by slow waves. Thus, it is possible that the functional modulation in the high beta and gamma bands between regions may be altered in schizophrenia.

We could not investigate the connectivity analysis in every region of the DMN because the connectivity between the close regions included the effects of volume conduction or crosstalk. In this study, two key regions, the PCC and MPFC, of the DMN were selected to estimate the connectivity analysis. Global connectivity analysis including more regions may be performed in future studies.

### DMN activity according to the frequency band

Although the beta band showed a smaller DMN activity than that of the alpha band, the DMN activity of the beta was also identified in the PCC. On the other hand, there was no positive DMN activity in the contrast maps of the theta and gamma bands. In a recent review of gamma synchrony, many studies reported elevated high-frequency EEG activity in schizophrenia patients [66]. But there are opposite results due to difference in sample size, imaging modality, or recording site [67]. Furthermore, a visual search task might increase the theta and gamma powers during the task. It is known that various tasks such as memory and attention lead to a power increase in the theta and gamma bands, together with a power decrease in the alpha and beta bands [68–70]. Thus, alpha and beta activation would occur during resting states, especially in the PCC and precuneus, while theta and gamma activation would occur during at-task states, especially in the MPFC.

### DMN activity at high frequency

The regional mapping in this study using MEG does not completely reproduce the DMN regions of fMRI studies. Nevertheless, future studies examining the DMN activity of high frequency oscillations are important. Because neural activity changes very quickly, neuronal activity faster than the BOLD change will help us to understand the brain’s responses during a task, which induces fast brain oscillation, as well as at rest. A single frequency band has a different DMN aspect from another frequency band. Very slow BOLD fluctuation is different from fast activities such as in the theta, alpha, beta, and gamma bands. Thus, many studies have examined the correlation between BOLD and EEG using simultaneous recording [71, 72]. Because MEG cannot be simultaneously measured alongside fMRI, combination studies of fMRI and EEG would complement the missing links of the DMN regions between fMRI and the present study.

The majority of the DMN studies used a memory paradigm for a goal-oriented task. In the present study, we used a different goal-oriented task that involved searching for Wally on a visually presented figure. A limitation of our study is the effect of eye movements during a visual search task. To minimize the eye-movement effect, we manually removed the time intervals that exhibited excessive eye movements. In addition, a working memory paradigm may produce different results from our study, especially on MPFC, since the prefrontal cortex is related to working memory [73]. For example, a working memory study may result in deactivation in the MPFC during the task. However, many studies have reported that various different experiments consistently show a similar DMN in oddball experiments [74] and visual search experiments [75]. Further studies are required to investigate the DMN activity in MEG with various tasks.

Another limitation is antipsychotic medications for schizophrenia patients, which may influence the results. Previous research on the relationship between antipsychotic dose and neural modulation using fMRI suggests that antipsychotics diminish neural activation in motor and default mode networks in schizophrenia patients [76]. Nevertheless, our study demonstrated augmented activity in schizophrenia. Further research will be necessary to uncover more evidence for the medication effect on the DMN activity in schizophrenia.

### Effect of the DMN activity in schizophrenia patients

The DMN activity in the PCC was significantly enhanced in schizophrenia patients (Figures 2 and 4). These data partly support a previous schizophrenia DMN study using fMRI [8]. In that study, schizophrenia patients showed greater DMN activation in the posterior and anterior cingulate and the middle and superior frontal gyri compared with the healthy controls. Compared with the fMRI study, our study did not show the DMN difference between two groups in the anterior cingulate and MPFC. However, the power spectrum of the schizophrenia group at rest showed a prominent increase in the MPFC and the PCC. We also found a significant correlation between DMN abnormality and symptoms in the MPFC. Previous studies showed that the abnormal DMN activity in schizophrenia is related to schizophrenia symptoms [3, 8]. It is known that abnormal fMRI activation of the anterior cingulate is correlated with working memory deficits in schizophrenia patients [5], and relates to an inability to correctly modulate internal thoughts and task processing, which may play a role in the positive symptoms of schizophrenia [8]. Therefore, the MPFC would be a region that reflects clinical symptoms, while the PCC is a region that can be used to discriminate between schizophrenia patients and healthy participants. fMRI and MEG show a similar relationship between clinical symptoms and DMN activity, which implies that the DMN activity in the gamma band is related to the slow BOLD fluctuation. However, the gamma band activity does not show a significant DMN difference between schizophrenia and control groups. In addition, DMN activities in the alpha and beta bands are not correlated with clinical measures. The relatively small samples used in this study make it difficult to observe weak to moderate effects. Further studies with larger samples are needed to replicate and extend the present findings.

In this study, resting-state activity in schizophrenic patients is significantly different from that in controls. However, this resting-state abnormality is not correlated with clinical measures. Resting-state activity can still be used to diagnose schizophrenia because it is a task-free and simple experiment.

Furthermore, our study showed the abnormal DMN and resting-state activity in the PCC and MPFC. There are various techniques to modulate specific brain regions by invasive and non-invasive stimulation. In the future, modulation of abnormal DMN activity at rest may provide a novel therapeutic approach.

## Conclusions

We used MEG to find that the DMN regions were mainly in the alpha band. Additionally, resting and DMN activities were augmented in the PCC in schizophrenia patients. Furthermore, the coherence between the PCC and MPFC was decreased in the gamma band at rest in schizophrenia.
